# Evolution of the multifaceted eukaryotic *akirin *gene family

**DOI:** 10.1186/1471-2148-9-34

**Published:** 2009-02-06

**Authors:** Daniel J Macqueen, Ian A Johnston

**Affiliations:** 1 Gatty Marine Laboratory, School of Biology, University of St Andrews, St Andrews, Fife, KY16 8LB, UK

## Abstract

**Background:**

Akirins are nuclear proteins that form part of an innate immune response pathway conserved in *Drosophila *and mice. This studies aim was to characterise the evolution of *akirin *gene structure and protein function in the eukaryotes.

**Results:**

*akirin *genes are present throughout the metazoa and arose before the separation of animal, plant and fungi lineages. Using comprehensive phylogenetic analysis, coupled with comparisons of conserved synteny and genomic organisation, we show that the intron-exon structure of metazoan *akirin *genes was established prior to the bilateria and that a single proto-orthologue duplicated in the vertebrates, before the gnathostome-agnathan separation, producing *akirin1 *and *akirin2*. Phylogenetic analyses of seven vertebrate gene families with members in chromosomal proximity to both *akirin1 *and *akirin2 *were compatible with a common duplication event affecting the genomic neighbourhood of the *akirin *proto-orthologue. A further duplication of *akirins *occurred in the teleost lineage and was followed by lineage-specific patterns of paralogue loss. Remarkably, *akirin*s have been independently characterised by five research groups under different aliases and a comparison of the available literature revealed diverse functions, generally in regulating gene expression. For example, *akirin *was characterised in arthropods as *subolesin*, an important growth factor and in *Drosophila *as *bhringi*, which has an essential myogenic role. In vertebrates, *akirin1 *was named *mighty *in mice and was shown to regulate myogenesis, whereas *akirin2 *was characterised as *FBI1 *in rats and promoted carcinogenesis, acting as a transcriptional repressor when bound to a 14-3-3 protein. Both vertebrate Akirins have evolved under comparably strict constraints of purifying selection, although a likelihood ratio test predicted that functional divergence has occurred between paralogues. Bayesian and maximum likelihood tests identified amino-acid positions where the rate of evolution had shifted significantly between paralogues. Interestingly, the highest scoring position was within a conserved, validated binding-site for 14-3-3 proteins.

**Conclusion:**

This work offers an evolutionary framework to facilitate future studies of eukaryotic *akirins *and provides insight into their multifaceted and conserved biochemical functions.

## Background

Akirin is a recently discovered protein with an essential function in the *Drosophila melanogaster *immune deficiency (Imd) pathway, which responds to gram-negative bacterial infection [1]. Akirin was strictly localised to the nucleus and acted in concert with Relish (a fly homologue of the vertebrate NF-kB transcription factor) to induce the expression of a subset of downstream pathway components [1]. The knockdown of the fly *akirin *gene caused a lethal embryonic phenotype [1]. *akirin *is conserved in vertebrates as at least two genes that were named *akirin1 *and *akirin2 *[1]. In mice, *akirin2 *functions in the toll-like receptor (TLR), tumour necrosis factor (TNF) and interleukin (IL)-1β signalling pathways, again at the level of/downstream of NF-kB to induce the transcription of several immune-response genes including the anti-inflammatory cytokine interleukin-6 (*IL-6*) [1]. The knockout of the individual mammalian *akirin *copies produced distinct phenotypes; whereas *akirin1*^-/- ^mice had no obvious phenotype, ablation of the *akirin2 *gene was embryonic-lethal [1]. Thus, seemingly, the role of invertebrate *akirin *in embryonic development and the innate immune response is most strongly conserved in *akirin2 *and *akirin1 *may have diverged in function [1]. While it is clear that vertebrate *akirin 1 *and *2 *are closely related, it is unknown whether they form part of a larger gene family related by gene duplication. Further, the exact origin and evolutionary relationships of *akirin1 *and *akirin2 *are not established.

In this paper we provide a detailed examination of the evolution of the *akirin *gene family in eukaryotes. Using an exhaustive computational screen including non-model species, we show that a single *akirin *proto-orthologue is highly conserved across invertebrate metazoans in terms of genomic organisation and coding features and identify orthologues in several more basal eukaryotes. Robust phylogenetic analysis revealed that *akirin *duplicated in a common chordate ancestor before the separation of jawed and jawless vertebrate lineages. We show that *akirin *genes have been characterised independently on several occasions, and suggest that a single, simple nomenclature system is employed in future studies. By bringing together the available *akirin *literature and examining the divergent molecular evolution of Akirin1 and 2 coding sequences, we provide significant insight into the multiple functions of this small gene family. A common feature of Akirins is to regulate gene transcription in several characterised signalling pathways, seemingly through interactions with intermediary factors such as 14-3-3 proteins.

## Results and Discussion

### *akirin *nomenclature

Future studies of *akirin *genes would benefit from a common nomenclature system to aid the dissemination of results between different research groups. Of the current names utilised, we suggest that the naming system employed by Goto et al. [1] is used in future submissions, since it is derived from the most detailed functional analysis and suitably describes the evolutionary relationships of different orthologues and paralogues. The designation '*FBI1' *[2] (i.e. for *akirin2*) is also founded on important functional data, but is very similar to a gene named factor binding IST protein 1 (*FBI-1*: **NP_056982**) and as with the name '*Mighty' *[3] (i.e. *akirin1*), does not account for evolutionary relationships within the gene family.

### The origin of *akirin *predates the metazoan lineage

**Figure 1:**
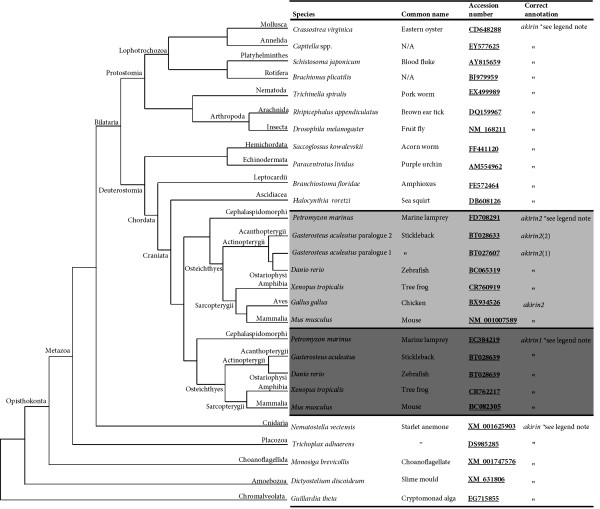
***akirin *genes were retrieved from an exhaustive computational screen of eukaryotic genome and transcriptome databases**. Representative sequences are mapped onto a cladogram demonstrating their phylogenetic relationships and branches are annotated with taxonomic information about separate clades. The branching of non-metazoan taxa is adapted from [5]. The branching of metazoan taxa is as previously demonstrated [7,8,10]. The split of *akirin* into two vertebrate clades was inferred from phylogenetic results of this study. More detailed information, including further taxa where sequences were retrieved can be found in additional file 1. * Note that *akirin *genes have been characterised under four other aliases: *subolesin *[36,37](tick *akirin*), *bhringi *[32] (another name for fly *akirin*), *mighty *[3] (mouse *akirin1*) and *FBI1 *[2] (rat *akirin2*).

An *akirin *proto-orthologue was not identified in the Bacterial or Archeal genomes examined, which either excludes a prokaryotic origin, or means that insufficient phylogenetic signal remains to identify these ancient orthologues. The earliest eukaryotes in which an *akirin *gene was retrieved were the protist taxa Alveolata (in *Guillardia theta*) and Heterolobosea (in *Naegleria gruberi*) (fig. 1). The Alveolata and the Euglenozoa (which are phylogenetically closely related to the Heterolobosea, [4]) are thought to have arisen close to 2 billion years ago [5]. This places an origin for *akirin *prior to the split of animal/plant/fungi lineages. However, an *akirin *gene was not identified in any plant or fungal genome as previously noted [1], although it was represented in the Amoebozoa (in *Dictyostelium discoideum*) and the choanoflagellate *Monosiga brevicollis *(fig. 1), which branches as the closest known outgroup to metazoans [6]. The presence of an *akirin *gene was an exception rather than a rule in non-animal eukaryotic genomes. This is consistent with the independent non-functionalization of *akirin *in many eukaryotic lineages, before it obtained a fundamental function in the metazoans.

### Genomic organisation of eukaryotic *akirins*

**Figure 2:**
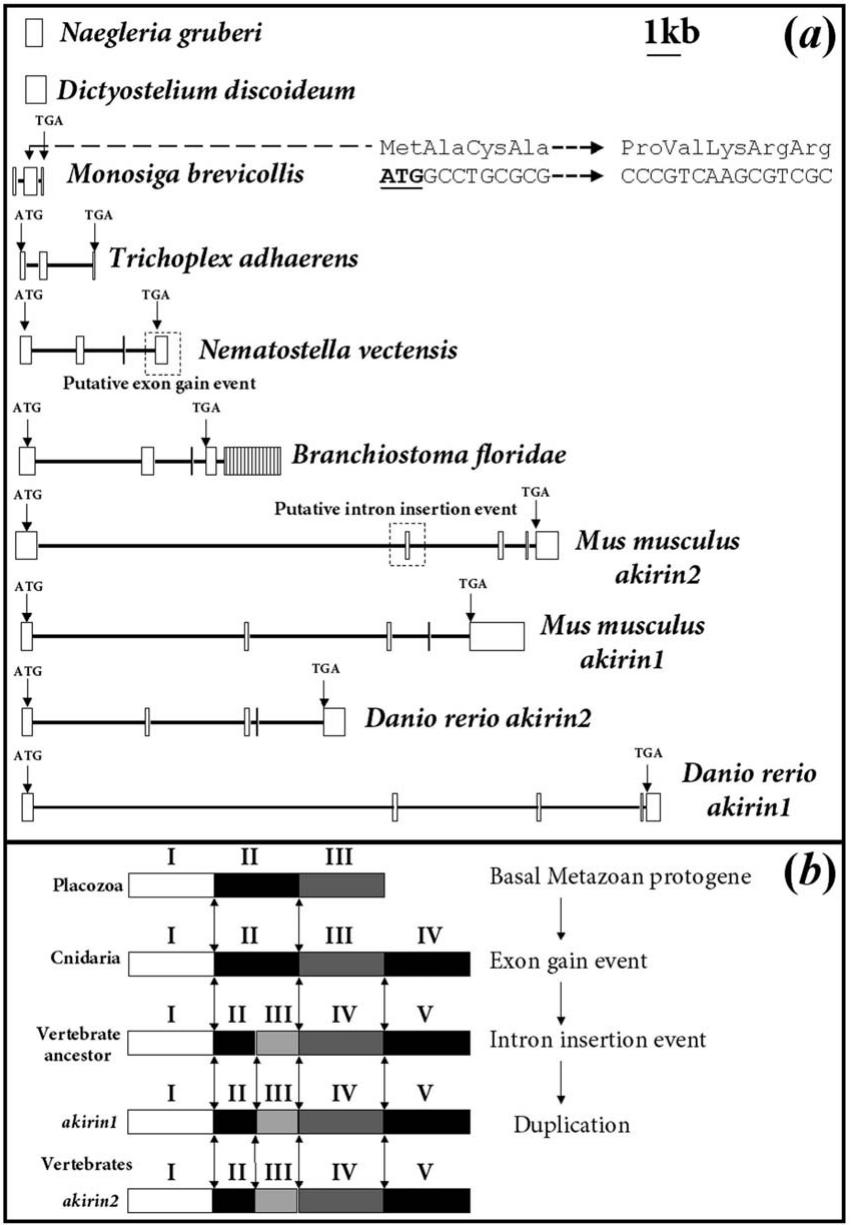
**(*a*). The genomic organisation of *akirin *orthologues across eukaryotic lineages**. The schematic diagram is made to scale with exons as white boxes and introns as black lines. Positions of start and stop codons are identified in metazoan exons. Evidence is also presented suggesting that the coding sequence of the metazoan *akirin *proto-orthologue was derived from exons 2/3 of the choanoflagellate (*M. brevicollis*) gene (see main text). *B. floridae akirin *is composed of 5 exons and exon 5 is shaded with vertical lines to show that it is not equivalent to exon 5 of vertebrate *akirins *(see main text). (*b*). Shows the conservation of exon-exon boundaries across the metazoans. Different exons are shaded in different colours and are numbered with roman numerals. Exons are not to scale and represent an archetypal genomic organisation for the taxa shown. Double ended-arrows indicate conservation of exon-exon boundaries. A scenario depicting the evolution of the genomic organisation of *akirin *genes is shown (also, see main text).

In *D. discoideum *and *N. gruberi*, *akirin *comprises a single exon, whereas *M. brevicollis *has conserved a 3-exon gene (fig. 2a). Choanoflagellates are the closest known living relatives to metazoans and its genes are comparably rich in intronic sequences [6]. Thus, relative to these more basal eukaryotes, it might be expected that certain features of the *M. brevicollis akirin *proto-orthologue would be conserved with animal *akirins*. While the two exon-exon boundaries of the choanoflagellate gene are not conserved with any metazoans (not shown), a string of 4 codons (ATG-GCC-TGC-GCG) 38 nucleotides into the second exon code the signature residues Met-Ala-Cys-Ala, conserved at the start region of all invertebrate metazoan Akirins (fig. 2a). Additionally, in close downstream proximity to this motif, a nuclear localization signal (NLS) (Pro-Val-Lys-Arg-Arg) is present (fig. 2a), which is conserved in metazoan sequences. These findings suggest that the metazoan *akirin1/2 *coding sequence was derived from exons 2 and 3 of a single gene in a common ancestor to metazoans and choanoflagellates.

A comparison of the genomic organisation of metazoan *akirins *provides insight into their evolutionary heritage (fig. 2). In all vertebrate species examined (mouse and zebrafish shown), *akirin1 *and *akirin2 *are organized as 5 exons of comparable size and 4 more variable introns (fig. 2a). In cephalochordates (*Branchiostoma floridae*),* akirin *also comprises 5 exons, although exon 5 is made up solely of untranslated nucleotides (fig. 2a, shaded in vertical lines). In fact, exon 4 of the *B. floridae *gene is equivalent to exon 5 of vertebrates (fig. 2a, evidenced by conserved position of stop codon) and the addition of exon 5 was probably a lineage specific acquisition. In Placozoans (*Trichoplax adhaerens*), *akirin *comprises 3 exons (fig. 2a). In Cnidarians (*Nematostella vectensis*), which represent a basal metazoan lineage that branched later than Placozoans [7,8], *akirin *comprises 4 exons. The boundary of exons 1/2 of Placozoan *akirin *is conserved with the boundary of exons 1/2 in all other metazoans examined (fig. 2b). Further, the boundary of exons 2/3 of Placozoan *akirin *is conserved with the boundaries of exons 2/3 in sea anemone/amphioxus and exons 3/4 in vertebrates (fig. 2b). Additionally, the boundary of exons 2/3 and 3/4 of the amphioxus/sea anemone genes are respectively conserved with the boundary of exons 3/4 and 4/5 in vertebrate *akirin1/2 *(fig. 2b). The most parsimonious evolutionary scenario to account for these distributions of conserved exon-exon boundaries is that firstly, an exon-gain event occurred in the *akirin *gene after the split of Placozoans with a common ancestor to Cnidarians and Bilatarians (fig. 2b). In support of this, exon 4 of the anemone/amphioxus proto-orthologue and vertebrate *akirin1/2 genes *starts with the last three residues of the protein (consensus sequence: Tyr-Val/Leu-Ser), which are conserved in all animals examined except Placozoans. Subsequent to the proposed exon gain event, an intron was seemingly inserted into exon 2 of *akirin *in a common deuterostome ancestor, after the split of cephalochordates and higher chordates, but before the event separating *akirin1 *and *akirin2 *(fig. 2a, b). We conclude that strong stabilising pressures have been enforced throughout metazoan evolution to maintain the comparable genomic organisation of present-day *akirin *genes across diverse taxa, in support of ancient patterns of gene regulation.

### Metazoan *akirin *genes

We performed an exhaustive search for *akirin*s in animal genomes and transcriptomes employing a broad taxonomic sampling strategy. These results are summarised in fig. 1 and additional file 1. In virtually all diploid vertebrates examined, a single *akirin1 *and *akirin2 *gene was identified. Almost without exception, both genes were strongly represented among EST databases of model and non-model vertebrate species. In common with the gnathostomes (jawed vertebrates), *Petromyzon marinus *(marine lamprey) had two sequences with marked identity to *akirin *(fig. 1, additional file 1). However, one was an EST that could not be identified in the Ensembl 5.9X genome pre-assembly and was partial at the C-terminal. Further, a single *akirin *orthologue was retrieved in the Myxinid (hagfish) lineage (additional file 1). In the model Avian *Gallus gallus *(red jungle fowl), no *akirin1 *orthologue was present in the current Ensembl genome assembly. Further, it was not represented among ~600,000 Genbank *G. gallus *ESTs, despite the presence of multiple positive *akirin2 *hits. Likewise, in other model birds including zebra finch (*Taeniopygia guttata*) and turkey (*Meleagris gallopavo*), no *akirin1 *orthologues were retrieved in EST databases containing ~92,000/17,500 respective sequences. Thus the absence of *akirin1 *in the class Aves reflects the genuine loss of a gene family member, rather than repeated artefacts of insufficient sequencing resolution. This is consistent with a recent finding showing that the number of gene family members common to tetrapods/teleosts is markedly reduced in the class Aves [9]. Interestingly, gene families, which, like *akirins*, had known roles in the immune system, were the most strongly affected [9].

In many invertebrate metazoans, a single gene was retrieved that shared significant identity to fly *akirin *and vertebrate *akirin1 *and *akirin2 *across its entire length (fig. 1, additional file 1), but had no clear identity to other characterised or uncharacterised genes. This included several bilaterian lineages with a strong representation of deuterostome and protostome taxa, plus more ancient phyla including Cnidarians and Placozoans, among the most ancient known animals [7,8]. However, an orthologue was not retrieved in sponges. A notable invertebrate lineage lacking an *akirin *gene was the family Cionidae, which has a completed high-resolution genome sequence and an abundance of EST sequences. This is consistent with the observation that the compact genome of *Ciona intestinalis *(~150 Mb) has undergone significant gene loss compared to other deuterostomes [10]. However, another Ascidian (*Halocynthia roretzi*) has retained an *akirin *orthologue.

### The *akirin *gene duplicated in a common chordate ancestor

**Figure 3:**
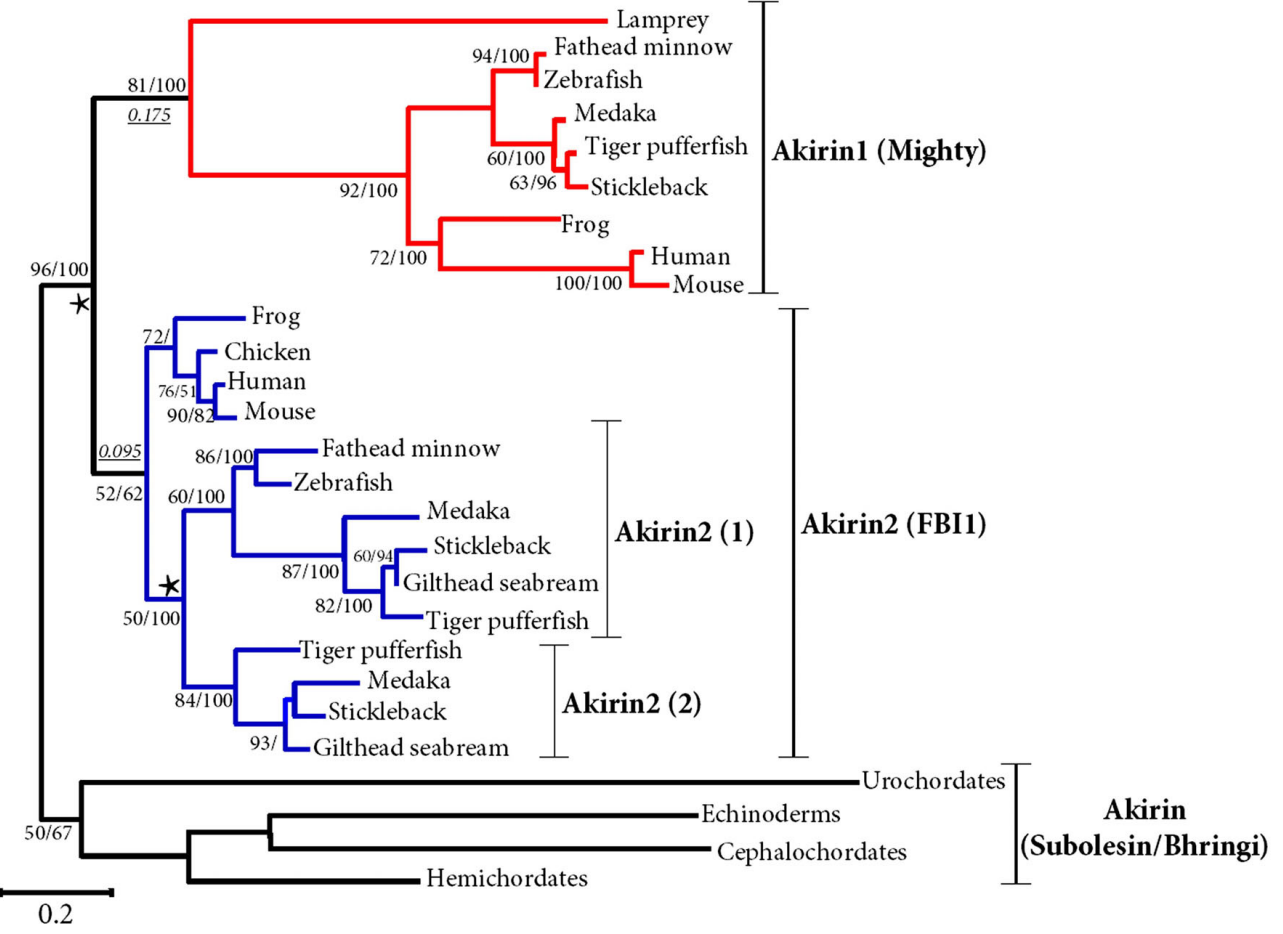
**ML tree depicting the phylogenetic relationships of vertebrate Akirin1 and Akirin2 proteins and the single Akirin protein of four invertebrate deuterostomes**. The corresponding amino acid sequence alignment is provided in additional file 2. The tree is rooted at the base of the invertebrate deuterostome clade. A corresponding Bayesian analysis was performed on the same dataset. Branch confidence values are shown as ML bootstrap values/Bayesian posterior probabilities. Both methods produced near identical topologies and a strongly supported branching of separate clades for Akirin1 (red branches) and Akirin2 (blue branches) is observed. Branch lengths leading to the Akirin1 and Akirin2 clades are shown underlined in italics and were derived from the ML analysis. The suggested nomenclature of the Akirin family, along with other known aliases (in brackets) is shown to the right of the tree. The scale bar shows the number of substitutions per site.

**Figure 4:**
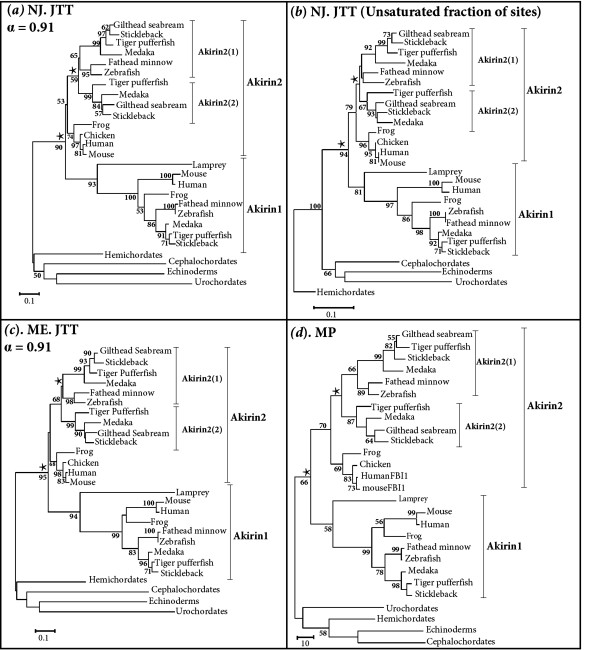
**Supporting phylogenetic reconstructions displaying the relationships of vertebrate Akirin1 and Akirin2 proteins and the single Akirin protein of four invertebrate deuterostomes**. The corresponding amino acid sequence alignment is as in fig. 3. (*a*) Is an unrooted NJ tree produced using the JTT model and implementing a gamma distribution parameter as estimated by ML. (*b*) Is a NJ tree reconstructed from solely the unsaturated fraction of sites, using the program ASATURA [11] with the JTT model. (*c*) Is an unrooted ME tree produced using the JTT model and implementing a gamma distribution parameter as estimated by ML. (*d*) Is an unrooted MP tree constructed using the close-neighbour-interchange method. Each analysis supported the topologies returned by ML and Bayesian approaches (fig. 3). Branch support values represent a percentage of 1000 bootstrap replicates.

Since a single *akirin *sequence was invariably retrieved in invertebrate/non-metazoan eukaryotes and diploid vertebrates had no more than two *akirin *homologues, we hypothesised that *akirin *was an ancestral proto-orthologue that gave rise to two *akirin *paralogues in a common vertebrate ancestor. This was tested by phylogenetic analysis using vertebrate Akirin1 and Akirin2 amino acid sequences and the single sequence representing four invertebrate deuterostome lineages as outgroups. The sequence alignment is provided in additional file 2. Maximum likelihood (ML) and Bayesian phylogenetic analyses were initially performed (fig. 3). Further, to test the robustness of the analysis to different methods of reconstruction, we also performed neighbour joining (NJ), minimum evolution (ME) and maximum parsimony (MP) analyses (fig. 4). Additionally, a NJ tree was constructed solely from unsaturated positions in the alignment [11], to test the hypothesis that mutational saturation at certain sites might influence tree topology. Very similar topologies were retrieved by all approaches (fig. 3 fig. 4) and a clear branching was apparent internal to invertebrate outgroups, separating Akirin1 and Akirin2 clades (fig. 3, fig. 4, 96/90/94/95/66 % bootstrap confidence values by ML/NJ/'unsaturated' NJ/ME/MP and 100% posterior probability in Bayesian analysis). In both Akirin clades, vertebrate sequences branched according to established taxonomic relationships, including the expected split of the Sarcopterygii and Actinopterygii (fig. 3, fig. 4). The included lamprey sequence (**EC384219**) branched as the most external node of the vertebrate Akirin1 clade in all trees (fig. 3, fig. 4). The partial lamprey sequence that was not included in the phylogenetic analysis, shares higher sequence identity to *akirin2 *(41%), than to *akirin1 *(30.5%) and could represent a lamprey *akirin2 *orthologue. The single hagfish orthologue was not used in the final phylogenetic analysis, since in preliminary reconstructions, its position was unstable and it formed either a weakly supported branch as the most external node of the Akirin2 clade or alternatively, branched with more basal invertebrate outgroups. Taken together, these results indicate that Akirin1 and Akirin2 are indeed paralogues that arose by gene duplication in a common ancestor to the vertebrate lineage, prior to the separation of gnathostome and agnathan lineages.

### Conserved synteny between *akirin1 *and *akirin2 *containing chromosomal tracts

**Figure 5:**
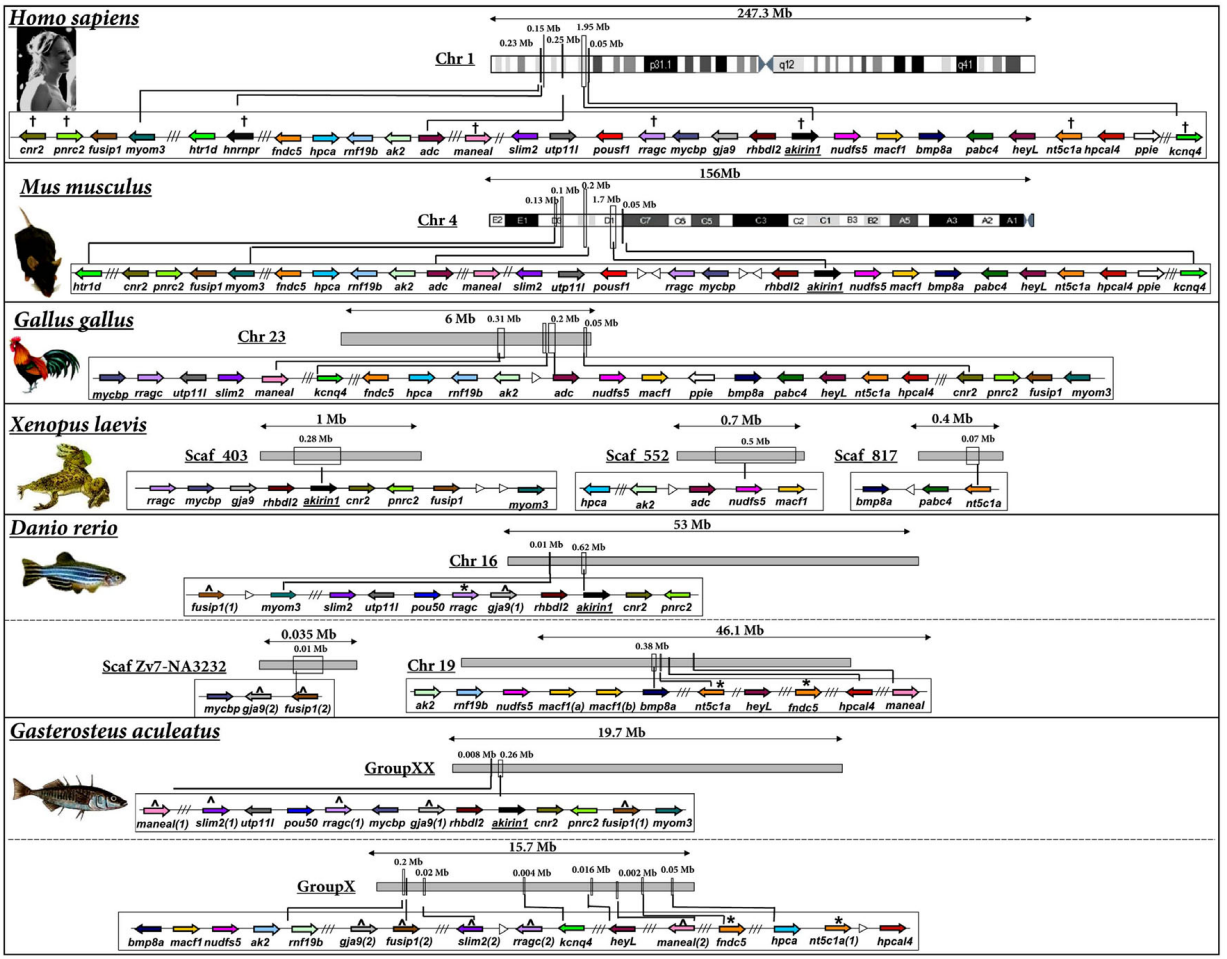
**The genomic neighbourhood surrounding *akirin1 *of mammals, birds, amphibians and teleosts**. Human genes are named as by the Human Genome Naming Consortium (HGNC) and orthologues are shown as identical coloured arrows, indicating the direction of transcription. The distance between genes is not to scale although approximate locations of chromosomal regions are identified. White arrowheads show a gene with no orthologue in other species in the diagram. Double and triple diagonal lines indicate a genomic distance respectively spanning 2 and >3 genes. Crosses on the human tract identify genes that share a closely related gene family member on the chromosomal tract containing *akirin2 *(please see corresponding crosses on fig. 6). Robust phylogenetic reconstructions of evolutionary relationships within these gene families were also established (see fig. 7). ^ indicates teleost genes where both paralogues are conserved on duplicated chromosomal tracts within the scale of the diagram. * indicates teleost genes where a paralogue was identified located outside of the scale of the diagram. Clear double conserved synteny is present in teleost genomes relative to diploid vertebrate relatives.

**Figure 6:**
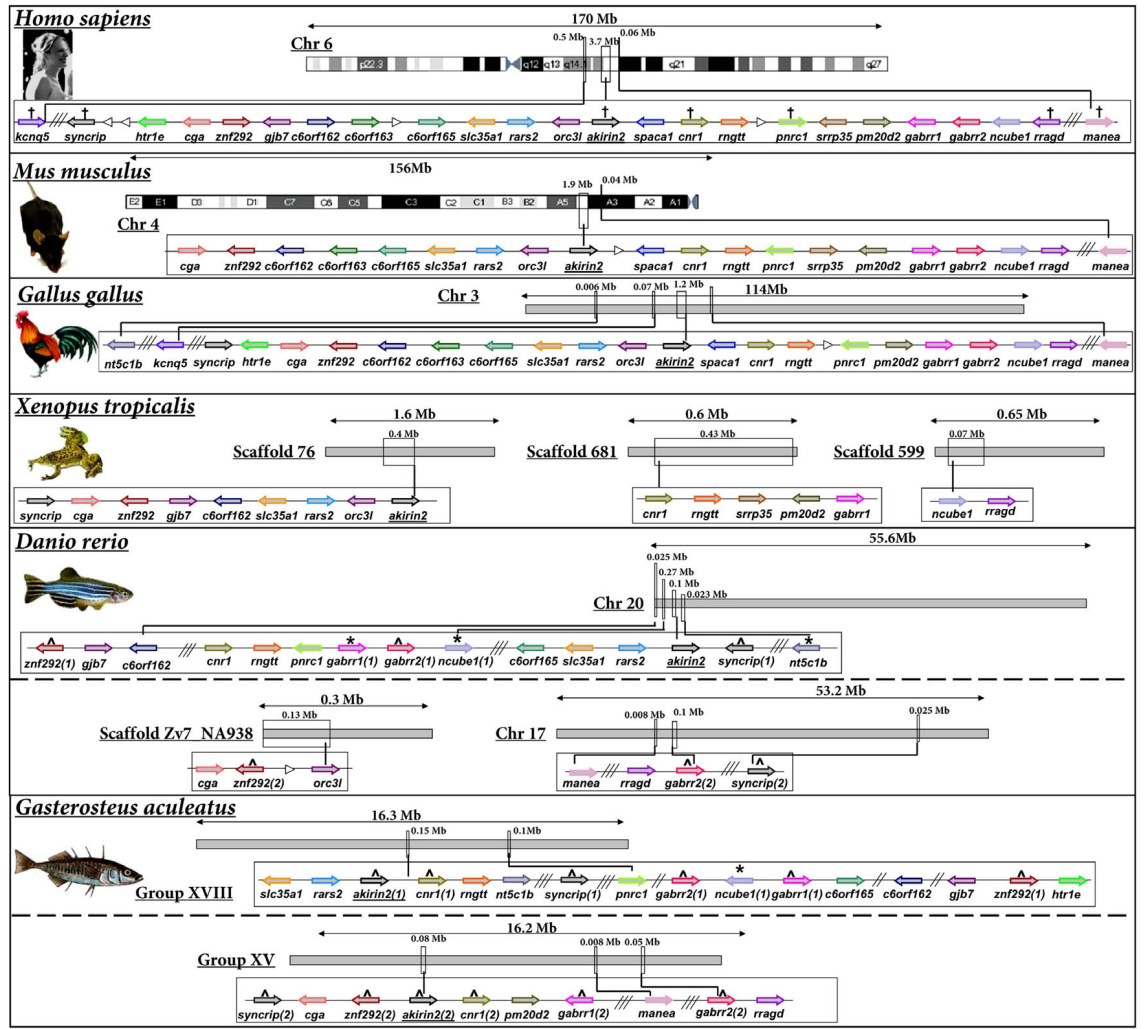
**The genomic neighbourhood surrounding *akirin2 *of mammals, birds, amphibians and teleosts**. Details are as in the fig. 5 legend.

Next, we examined the genomic neighbourhood surrounding *akirin1*/*2 *in mammals, birds, amphibians and fishes (fig. 5, fig. 6). In all vertebrate genomes examined, *akirin1 *and *akirin2*, when both present, were positioned on different chromosomes, except in mice, where they are located on different ends of chromosome 4, separated by ~100 Mb. A comparison of genes in the neighbourhood of *akirin1 *and *akirin2 *(i.e. separately considering fig. 5. and fig. 6) demonstrates a strong level of conserved synteny across vertebrate classes, with limited intra-chromosomal rearrangements and few inter-chromosomal rearrangements in tetrapod species with known karyotypes. In teleosts, two tracts were present with conserved synteny relative to single *akirin1/akirin2-*containing regions of tetrapods (see following section). Consistent with BLAST homology screens (fig. 1, additional file 1), an *akirin1 *gene was absent from the *G. gallus *genome and gene order was disrupted around this region relative to mammalian and amphibian genomes examined (fig. 5). In summary, these results indicate that the chromosomal organisation of *akirin-*containing tracts of vertebrate genomes were conserved from a common ancestor prior to the speciation events separating the major vertebrate classes.

### *akirins *and the teleost whole genome duplication event

A single *akirin1 *gene was identified in all teleost species examined, whereas two *akirin2 *copies were retrieved from Acanthopterygian taxa i.e. pufferfishes, medaka, sticklebacks and sea bream. All methods of phylogenetic analysis separated teleost *akirin2 *sequences into two clades (fig. 3, fig. 4). The first was represented by one of the two sequences in species of the Acanthopterygii and the single Ostariophysi copy (i.e. zebrafish, *Danio rerio *and fathead minnow, *Pimephales promelas*) (fig. 3, fig. 4) The second clade was represented by the remaining Akirin2 sequences of Acanthopterygian species (fig. 3, fig. 4). Thus, each tree branches prior to the split of Acanthopterygian and Ostariophysian samples, which indicates that this duplication event occurred in a common teleost ancestor rather than in the Acanthopterygian lineage. However, statistical confidence in this branching was weak by all methods (fig. 3, fig. 4, 50/59/<50/68/<50% respective bootstrap support in the ML/NJ/'unsaturated' NJ/ME/MP analyses) excepting the Bayesian analysis (fig. 3, 100% posterior probability values). Bayesian phylogenetic reconstruction was shown under certain conditions to produce an overestimate of branch confidence [12]. Thus, we also sought evidence to either provide support or refute this branching topology, using comparisons of conserved genomic synteny. The synteny map indicates that an expansive genomic region containing *akirin2 *duplicated in a common ancestor to zebrafish and stickleback (*Gasterosteus aculeatus*), since two orthologous chromosomal tracts exist in both species that retain common synteny to a single region in tetrapod genomes (fig. 6). Specifically, tetrapod genes are present in teleosts as either single orthologues interspersed between the two tracts (e.g. *rars2, rragd*, *pnrc1, rngtt*, *orc3l*, *gjb7*) or are present as duplicated co-orthologues on both regions (e.g. *akirin2*, *gabrr1*, *gabbr2*, *znf292*, *syncrip*) (fig. 6). A similar pattern of double conserved synteny is seen in teleosts relative to tetrapods on the *akirin1 *synteny map, although *akirin1 *is only retained on a single chromosome (fig. 5). These patterns of synteny may be the result of a genome tetraploidization event that occurred in a basal teleost ancestor after the split of the Actinopterygii and Sarcopterygii lineages [13,14]. However, this interpretation requires that one of the *akirin1 *paralogues from this event was non-functionalised either in a common teleost ancestor, or within individual lineages. Furthermore, one of the *akirin2 *paralogues must have been non-functionalised in an ancestor to the Ostariophysi lineage, since a single *akirin2 *gene is found in zebrafish and fathead minnow.

Duplicated genes from teleost species are generally annotated as either gene-1/gene-2 or gene-A/gene-B according to the order of their discovery. However, this nomenclature system is rarely based on phylogenetic premises and generally does not accommodate paralogues from distinct duplication events in different teleost lineages. For certain genes where teleost duplicates have been retained from both the teleost WGD and more recent lineage specific events, appropriate nomenclature systems have been proposed to simplify confusing existing naming systems (e.g. MyoD: [15]). Due to the fact that *akirins *are uncharacterised in fishes, we have a rare opportunity to set out a logical nomenclature framework from the onset of their study. We recommend, as indicated in fig. 1 and additional file 1, that teleost *akirin2 *paralogues derived from the teleost whole-genome duplication event [13,14] are named as either *akirin2*(1) or *akirin2*(2). Paralogues of these genes from more recent duplication events in certain teleost lineages e.g. salmonids [16] should be named *akirin2*(1a/1b) or *akirin2*(2a/2b). Similarly, if new teleost *akirin1 *paralogues are discovered in the future then an equivalent naming system should be employed.

### Phylogenetic analysis of gene families present on both *akirin1 *and *akirin2 *containing chromosomal tracts

**Figure 7:**
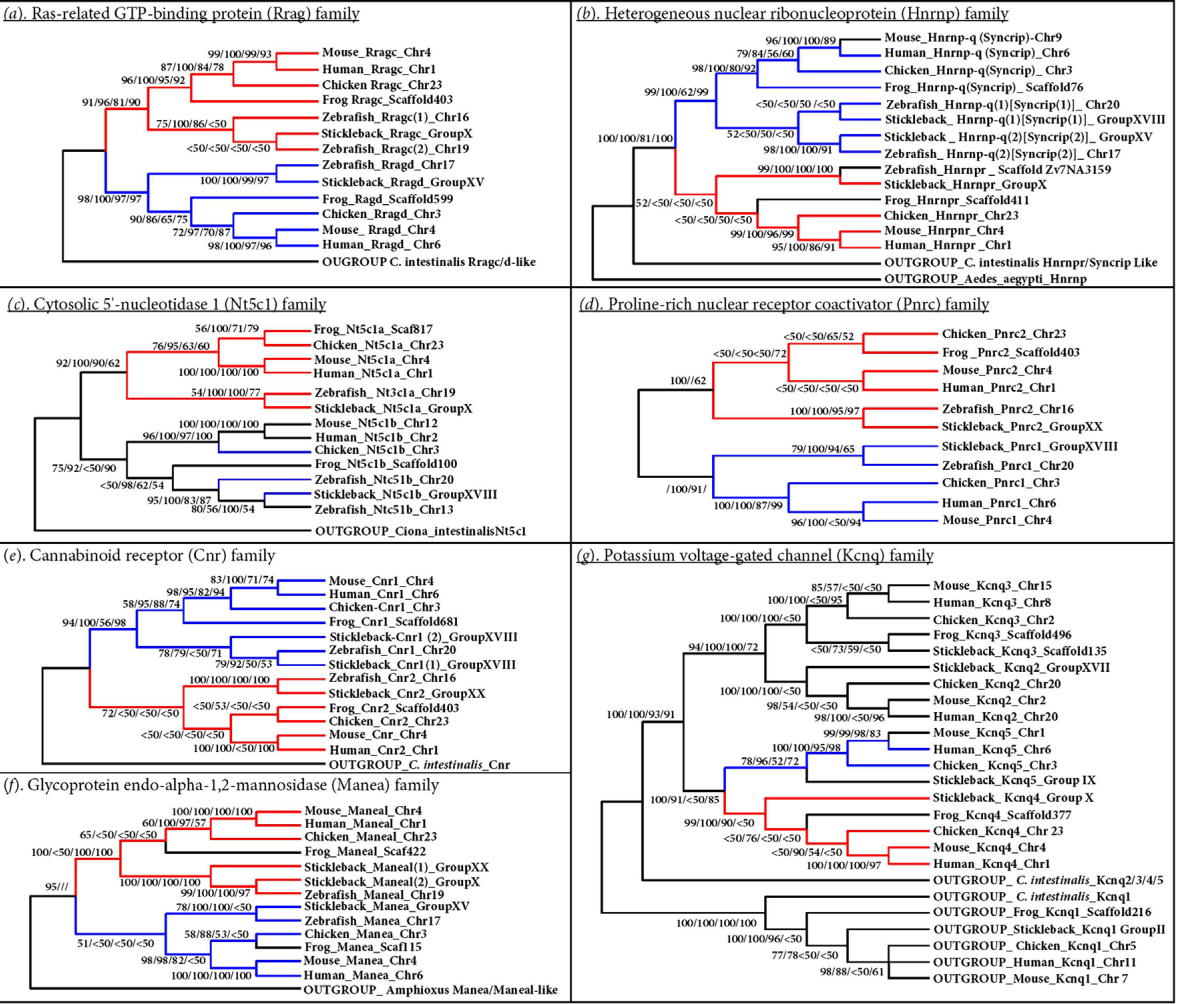
**Phylogenetic reconstruction of vertebrate gene families with members in chromosomal proximity to both *akirin1 *and *akirin2***. The corresponding amino acid alignments employed are provided in additional file 3. The shown topologies (*a-g*) were obtained by NJ and unless mentioned otherwise in the main text, were comparable to those produced by Bayesian, ML and MP analyses. Branch confidence values greater than 50% from each approach are shown in the order NJ/Bayesian/ML/MP. The ras-related GTP-binding family (*rragc *and *rragd*) (*a*), heterogeneous nuclear ribonucleoprotein family (*hnrnp-r *and *hnrnp-q *[HGNC name-*Syncrip*]) *(b*), cytosolic 5'-nucleotidase 1 family (*nt5c1a *and *nt5c1b*) (*c*), proline-rich nuclear receptor coactivator family (*pnrc1 *and *pnrc2*) (*d*), cannabinoid receptor family (*cnr1 *and *cnr2*) (*e*) and glycoprotein endo-alpha-1,2-mannosidase family (*manea *and *maneal*) (*f*) each contained two members in all non-teleost vertebrate genomes examined and were, with limited exceptions, present in syntenic chromosomal regions (or double conserved syntenic regions in some teleosts- see fig. 5 and fig. 6) where one family member was located near *akirin1 *and the other near *akirin2*. The potassium voltage-gated channel (*kcnq*) family (*Kcnq1/2/3/4/*5) (*g*) contains two family members (*kcnq4 *and *kcnq5*) that are, in most vertebrates, in genomic proximity to *akirin1 *and *akirin2 *respectively. Further details about each phylogenetic analysis are provided in the text. To summarise, the branching patterns for each of these families was compatible with a duplication event in the vertebrate lineage of chordates and was also concomitant to their genomic location in relation to *akirin1 *(indicated by red branches) and *akirin2 *(indicated by blue branches).

If *akirin1 *and *akirin2 *arose from the duplication of a region of the genome in a common vertebrate ancestor (either through a segmental duplication or tetraploidization), then other genes or gene families would have been duplicated at this time. Several vertebrate gene families are located in syntenic chromosomal regions where different members are located proximally to both *akirin1 *and *akirin2 *or to tracts where copies of these genes have been lost (i.e. in certain regions of double conserved synteny in teleosts, fig. 5, fig. 6 and on the region of chicken chromosome 23 shown in fig. 5). Several form small gene families, with just two members in diploid vertebrates (as observed for the *akirins*) and where one member is found on each chromosomal tract containing either *akirin1 *or *akirin2*. For example, in several vertebrate classes, genes for *rragc*, *nt5c1a*, *cnr2*, *pnrc2*, *hnrnp-r*, and *maneal *are found in the *akirin1 *genomic neighbourhood and have a single putative paralogue on the *akirin2 *chromosomal tract (respectively: *rragd*, *nt5c1b*, *cnr1, pnrc2*,*hnrnp-q *[aka *syncrip*] and *manea*) (compare fig. 5 and fig. 6). Comprehensive phylogenetic reconstructions of these gene families were performed with amino acid alignments using NJ, Bayesian, ML and MP approaches (fig. 7, alignments provided in additional file 3). For 5 of these 6 gene families, a single putative orthologue was identified in *C. intestinalis/B. floridae *genomes and employed as an outgroup. For Ras-related GTP-binding protein (Rrag), Heterogeneous nuclear ribonucleoprotein (Hnrnp), Cytosolic 5'-nucleotidase 1 (Nt5c1) and Proline-rich nuclear receptor coactivator (Pnrc) protein families, tree topologies were very similar by all methods of reconstruction (fig. 7a–d). In each case the tree branched into two clades containing family member orthologues from different vertebrate classes, closely reflecting expected taxonomic relationships (fig. 7a–d). Each of these branching topologies is compatible with a duplication event at the base of the vertebrate lineage, as shown for *akirins *(fig. 3, fig. 4).

Phylogenetic analysis of the Cannabinoid receptor (Cnr) family was sensitive to the reconstruction method and only the NJ analysis split the tree into two clades of Cnr1 and Cnr2 orthologues (fig. 7e). Other methods strongly supported a single Cnr1 clade, but did not resolve Cnr2 sequences into a single clade, when teleost sequences were included (not shown). However, it is noteworthy that previous phylogenetic studies have suggested that Cnr1 and Cnr2 (also known respectively as CB1 and CB2) duplicated from a single proto-orthologue in the vertebrate stem of the chordate lineage [17,18].

For the glycoprotein endo-alpha-1,2-mannosidase family, all four methods of reconstruction produced similar topologies in which the tree did not branch into separate Manea and Maneal clades due to the inclusion of teleost Manea sequences as the external branch of a clade containing solely other vertebrate Maneal sequences (not shown). We tested the hypothesis that tree topology was being influenced by mutational saturation at a proportion of sites in the alignment. When saturated positions were removed from the analysis, a NJ topology was obtained splitting the tree into separate vertebrate Manea and Maneal clades (fig. 7f). Therefore, it is possible that mutational saturation caused an aberrant branching of teleost Manea sequences and that the corrected tree again reflects a duplication event of a Manea/Maneal proto-orthologue in a common vertebrate ancestor.

In most vertebrate classes, two members of the potassium voltage-gated channel family (*kcnq4 *and *kcnq5*) were located in the respective genomic neighbourhood of *akirin1 *and *akirin2 *(fig. 5, fig. 6). This gene family contains up to five members in diploid vertebrates and 2 members in the *C. intestinalis *genome. All methods of phylogenetic analysis produced near identical topologies with a clade including vertebrate and *C. intestinalis Kcnq1 *orthologues that branched externally to remaining family members (fig. 7g). Internal to this clade, the other *C. intestinalis *Kcnq sequence branched externally to the remaining four vertebrate Kcnq sequences, which split into two well-supported clades containing Kcnq2/3 and Kcnq4/5 sequences respectively (fig. 7g). These clades split into sub-clades containing individual Kcnq2 and 3 orthologues and Kcnq4 and 5 orthologues (fig. 7g). This branching pattern can be explained by two duplication events in the vertebrate lineage, where a single proto-orthologue to *Kcnq2/3/4/5*, duplicated to create two ancestor genes to *Kcnq2/3 *and *Kcnq4/5 *which both duplicated again to produce *Kcnq2*, *kcnq3*, *kcnq4 *and *kcnq5 *genes as conserved in current vertebrate genomes.

The branching patterns of these gene families, are therefore generally consistent, not only with at least one duplication event in a common ancestor to mammals, birds, frogs and fishes, but in the case of the highlighted members, often reflect their respective chromosomal proximity to *akirin1 *or *akirin2*. In other words, when orthologues from a gene family (i.e. one clade in the tree) were located in the genomic neighbourhood of either *akirin1 *or *2*, paralogues from that family (in the other clade) tended to be proximal to, or at least on the same chromosome as the other *akirin *copy. A parsimonious explanation for these findings is that a duplication event occurred in the vertebrate stem of the chordates that affected a chromosomal region containing both proto-orthologues to *akirin *and to components of neighbouring gene families. Two-rounds of genome polyploidisation in vertebrates has been long been proposed [e.g. [19]] and support for this hypothesis has been obtained by comparing vertebrate genome organisation, with deuterostome relatives with unduplicated genomes, including urochordates [20,21] and recently cephalochordates [10]. For example, Putnam et al. showed that Gnathostome genomes share quadruple conserved synteny with the *Branchiostoma floridae *genome providing 'conclusive evidence for two rounds of duplication on the jawed vertebrate stem' [10]. However, this idea has been historically controversial and certain studies using phylogenetic analysis of vertebrate gene families found a lack of supporting statistical evidence e.g. [22,23], while others found results compatible with the hypothesis e.g. [24].

### Test for selection and functional divergence after the Akirin duplication

It is widely accepted that gene duplication can create opportunities for functional divergence in paralogues. Divergence is thought to occur where one duplicate retains the original protein function and the other accumulates changes, (either through redundancy or by positive selection) or alternatively, through the partitioning of the functions of an unduplicated ancestor protein [reviewed in [25]]. Whatever the mechanism, if functional divergence has occurred between duplicated genes, then it should be observable as changes within their coding regions, since functionally important and non-functionally important residues should evolve under different constraints.

It is known that Akirin1 and Akirin2 differ in at least one function [1]. The branch length leading to the Akirin1 clade is extended relative to Akirin2 in all phylogenies, (fig. 3, fig. 4). This suggests that after the *akirin *duplication, Akirin1 evolved at a faster rate than Akirin2. This result was confirmed by significant relative rate test results for several vertebrate lineages (result not shown). To examine whether this difference in evolutionary rate was accompanied by altered selective constraints, we examined pairwise rates of synonymous (dS) and non-synonymous (dN) substitutions between Akirin1 and 2 for several vertebrate lineages. Two approaches were implemented: firstly, the likelihood method of Goldman and Yang [26] and secondly, the Nei-Gojobori approach [27]. Both results were comparable and low dN/dS ratios (<<1) were estimated when different vertebrate lineages were compared for Akirin1 and Akirin2 (additional file 4). Specifically, dN/dS ratios averaged from both methods, were ~0.14 for Akirin1 and ~0.09 for Akirin2. Thus, Akirin1 and Akirin2 proteins, as a whole, have evolved under comparably strict purifying selection.

**Figure 8:**
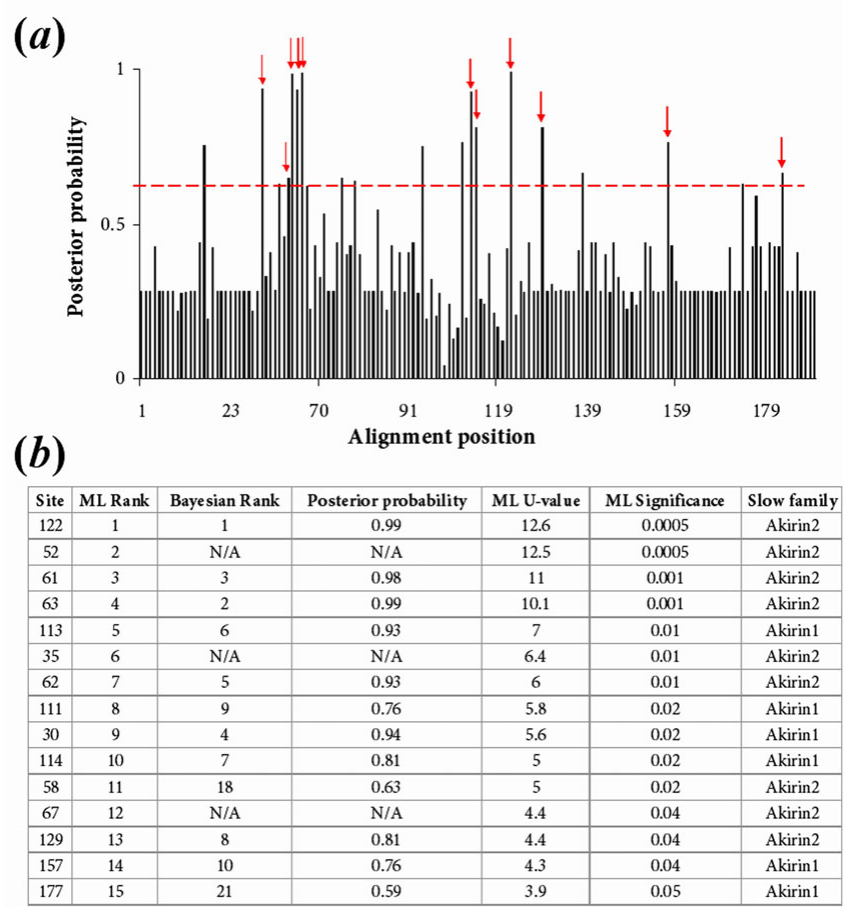
**(*a*). Site-specific profile predicting residues underlying type-I functional divergence between Akirin1 and Akirin2 paralogues measured by a Bayesian posterior probability method **[28]** in DIVERGE **[33]. After a cut-off value of 0.6 (dotted red line) was employed, ~20 sites were considered the most likely candidates for type-I divergence. Red arrows show sites that were also shown to be have evolved at a significantly faster/slower rate in one Akirin paralogue, using a ML LRT [29] (see part *b *of this figure). (*b*). Table summarising the 15 highest-ranking positions with significant rates shifts between Akirin1 and Akirin2 using the ML LRT [29]. Also shown are the ranking of these same sites, established using the posterior probability approach. N/A in the Bayesian rank column indicates that the test could not be performed at this position, since this method cannot accommodate alignment gaps. The two methods produced similar results and the top 10 Bayesian candidate sites for functional divergence were among the top 15 ranking sites by the ML approach.

**Figure 9:**
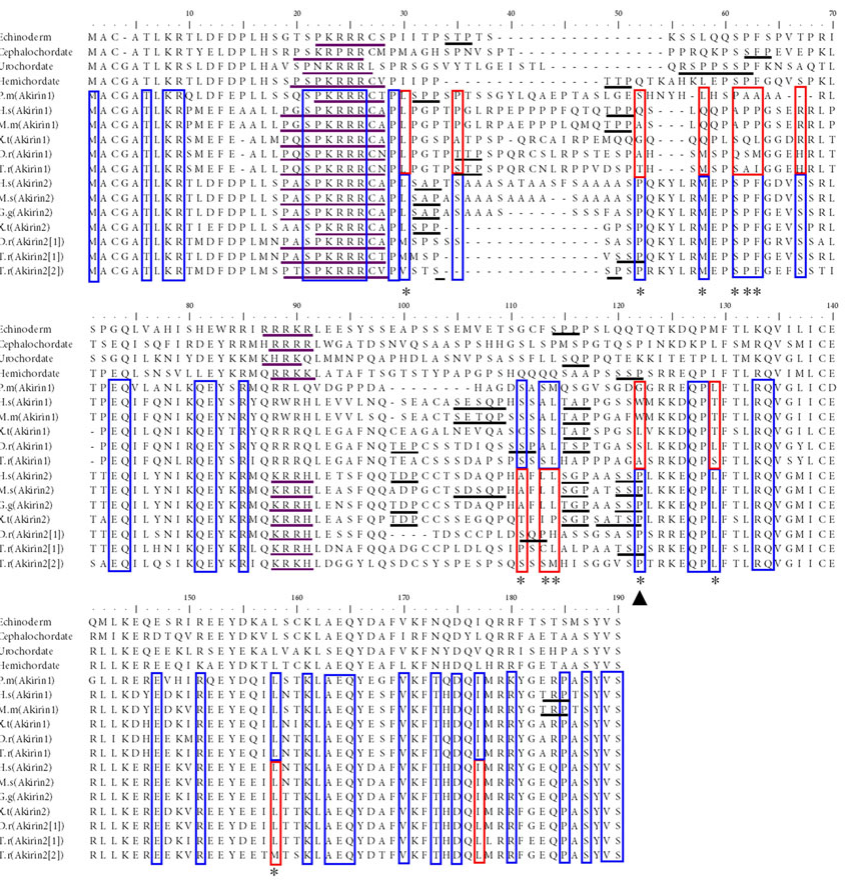
**Summary of ML LRT results for Akirin1 and Akirin2 proteins mapped onto an amino acid alignment also including four invertebrate deuterostome outgroups**. Sites that have evolved at the same rate in Akirin1 and 2, but at a significantly slower rate than the average for all sites, are boxed in blue. Sites with both blue and red shading correspond to positions that have evolved at either a significantly faster (red boxes) or slower (blue boxes) rate in one Akirin paralogue compared to the other. Significance is at the 5% level in all cases. Sites evolving at significantly different rates between Akirin paralogues, that were also significant in the DIVERGE [28,33] test, are highlighted with a star. The original LRT alignment output, with 27 Akirin genes is provided in additional file 5. Shown, underlined in purple and black respectively, are two NLSs and putative 14-3-3 binding sites (after [2]). The highest scoring site (position 122, marked with black arrowhead) in both tests was a proline conserved solely in Akirin2 orthologues and Akirin proto-orthologues, but not in Akirin1 proteins. This site falls within an experimentally validated 14-3-3-recognition site [2]. Alignment name abbreviations are: P.m (*P. marinus*), H.s (*H. sapiens*), X.t (*X. tropicalis*), D.r (*D. rerio*), T.r (*Takifugu rubripes*), G.g (*G. gallus*).

It is known from both a large-scale protein-protein interaction study [30] and specific studies [2,31,32] that the ancestor protein to Akirin1 and Akirin2 has many binding partners (>20 are currently known) and several of these are conserved in equivalent vertebrate pathways (see section: 'a synthesis of information on *akirin *function'). Thus, there are many potential residues of functional significance in Akirin (i.e. within putative binding sites) that may have evolved at different rates between Akirin1 and 2. Furthermore, binding sites for 14-3-3 proteins have been biochemically confirmed in Akirin2 [2]. Statistical methods have been proposed to predict whether functional divergence of related members of a protein family has occurred (e.g. [28]) and identify the most likely residues involved (e.g. [28,29]). Initially, we used a likelihood ratio test (LRT) implemented in the program DIVERGE [33] to test the hypothesis that functional divergence of Akirin1 and 2 was a reflection of a shift in evolutionary rates at certain amino acid sites between paralogues (known as type-I divergence, [28]). The model underlying this approach was described in [28]. To summarise, we tested whether the coefficient of functional divergence (*θ*) between Akirin1 and 2 clusters in the phylogenetic tree topology provided (as in fig. 3), was >0. The LRT predicted that functional divergence had occurred between Akirin1 and 2 (*θ *= 0.37 +/- 0.06, statistically significant [28,33]). Next, DIVERGE was used to establish the posterior probability of type-I divergence at each site in the alignment (fig. 8a). Employing a cut-off posterior probability value of 0.6, ~20 sites were predicted as the most likely candidate sites for type-I functional divergence. For comparative purposes, the same alignment and phylogeny was submitted to a ML LRT, which, like the Bayesian method provides a statistical framework where evolutionary rate shifts at particular protein positions can be established [29]. The statistically most likely positions predicted to underlie functional divergence were comparable by both methods, particularly for the highest-ranking candidates (fig. 8a, b). High scoring positions were mapped onto an amino acid alignment of the Akirin gene family, in relation to known functional motifs (fig. 9). A PDF output of the ML analysis, in its original format, is also provided (additional file 5).

The extreme N-terminus (first 30 residues) and C-terminus (last ~70 residues) of Akirin proteins are clearly under strong purifying selection based on the near absence of fast-evolving sites (additional file 5) and the presence of many sites that have evolved at a significantly slower rate than the average of all positions (fig. 9, additional file 5). Further, in these N and C-terminal regions, very few sites (respectively none and two) are predicted to contribute to functional divergence between Akirin1 and 2 (fig. 8, fig. 9). Of the last 65 sites in Akirins, 20% are conserved from basal metazoans to vertebrates and ~55% code for isofunctional replacements (not shown). Additionally, it is only the ~70 most C-terminal residues that share significant identify with the basal Amoebozoan and protist orthologues (not shown). Therefore these conserved regions must perform essential functions common to Akirins and are obvious candidates for experimental characterisation.

A known functional motif found in Akirins, is a highly conserved N-terminal NLS [1] (fig. 9). As expected, sites within this motif have evolved significantly slower than the average in all Akirins (fig. 9, additional file 5), in support of its necessity for nuclear localisation as demonstrated in insect and mammalian Akirins [1]. Further, another NLS was predicted in PSORT2 [34] to be present in Akirin of invertebrate deuterostomes (plus several other invertebrates, dating back to Placozoans, not shown) and Akirin2, but not Akirin1 (fig. 9). However, rate shifts at these sites were not predicted to contribute to functional divergence between paralogues. Interestingly, Akirin1 was detected in both the nucleus and cytoplasm of C2C12 myoblasts [3]. Further experimental tests will be needed to examine whether this second NLS augments the nuclear import of Akirin and Akirin2 proteins relative to Akirin1, which would have important implications for the sub-cellular context of the vertebrate paralogues.

Almost all of the highest scoring candidate positions for functional divergence between Akirin paralogues are found in the middle region of the protein (positions 30–130 in our alignment), which also has numerous sites that evolved at a significantly higher rate in both Akirin1 and 2 compared to the average of all positions (additional file 5). The highest scoring site for functional divergence in both the Bayesian analysis and ML LRT (site 122) corresponds to a proline conserved in all Akirin2 orthologues, two invertebrate Akirin orthologues but not in Akirin1 proteins (fig. 9). In all tetrapod and most teleost Akirin2 orthologues, as well as hemichordate Akirin, this site is the final residue of a putative 14-3-3-recognition site, biochemically validated in rodent Akirin 2 (consensus: serine/threonine -X-proline in rat Akirin2 [2]). Further, two other high scoring positions fall either on putative 14-3-3 binding sites (site 52) or are just upstream of a 14-3-3 binding site conserved in both Akirin1 and Akirin2 (sites 111 and 113–114). It is feasible that these sites have contributed to altered 14-3-3 binding properties of Akirin1 and 2. Another region that is a strong candidate for type-I divergence between Akirin1 and Akirin2 is found at sites 58–67. In this region, 5/10 positions have evolved at a significantly slower rate in Akirin2 than Akirin1 (fig. 8) and are among the highest scoring candidate residues for type-I functional divergence (fig. 9). This region may be a binding site that is functional in the invertebrate Akirins and Akirin2, but not in Akirin1.

### Putative 14-3-3 binding sites in Akirins

Of the five 14-3-3 protein-binding sites identified in rat Akirin2 [2], four are conserved across amniote orthologues (not shown), and fewer in teleost orthologues (fig. 9). Akirin1 has between one and four putative 14-3-3 binding sites across a broad phylogenetic range of vertebrates, generally in regions conserved with at least one Akirin2 protein. Deuterostome invertebrate Akirins generally have two to four 14-3-3 binding sites, usually in regions aligning with vertebrate Akirins, but rarely with other invertebrate Akirins (fig. 9). The *M. brevicollis*,* D. discoideum *and *N. gruberi *orthologues have a single putative 14-3-3 binding site whereas *G. theta *has none (not shown). Therefore, the number of potential 14-3-3 binding sites in Akirin proteins increased rapidly at the base of metazoan evolution. However, sites are absent or greatly reduced in certain metazoan lineages, including *D. melanogaster *(0 sites), *Anopheles gambiae *(0 sites), *Lumbricus rubellus *(0 sites) and *Caenorhabditis elegans *(1 site) (not shown). The preferred binding motifs of 14-3-3 proteins are Arg-Ser-x-Ser-x-Pro and Arg-x-x-x-Ser-x-Pro, although functional variations in these motifs are tolerated [35]. Almost invariably, sites in Akirin proteins have the consensus-binding site Ser/Thr-x-Pro or Ser-x-Ser/Thr-x-Pro (fig. 9). The single exception is the sea squirt sequence, which has a perfect site (Arg-Ser-Pro-Pro-Ser-Ser-Pro) (fig. 9). Unsurprisingly, multiple sites were needed for the formation of the Akirin2–14-3-3 complex [2]. Considering the variability in the number (sometimes none) and physical locations of 14-3-3 sites, it is likely that the binding affinity for 14-3-3 proteins will vary considerably between Akirin1 and Akirin2 paralogues within vertebrate species and between orthologues from different lineages.

### A synthesis of information on *akirin *function

In this section, we combine the findings of this study with available literature on the known roles of *akirin *genes in order to provide novel insight into their biochemical functions. We hope that this will prompt the sharing of *akirin *literature between researchers from different fields and open up new avenues of investigation.

Consistent with the embryonic lethal knockdown of *akirin *and *akirin2 *in flies and mice respectively [1], the ablation of *akirin *in the embryos of the nematode *C. elegans *by RNAi knockdown was also lethal ( search term: E01A2.6). Further, RNAi knockdown of *akirin *in ticks (i.e. *subolesin*, previously named 'protective antigen 4D8',[36]) dramatically affected the growth and fertility phenotype, with enormous associated reductions in survival, weight and oviposition, as well as developmental abnormalities in several different tissues [37]. These findings support the idea that *akirin *is an essential developmental gene across a broad phylogenetic range of metazoans. Another conserved feature of Akirins in metazoans is their nuclear localisation (fly: [1] and Flybase: ; mammals: [1-3] and broad or near-ubiquitous expression patterns in embryonic and adult tissues (fly: [1], nematodes: , search term: E01A2.6; ticks: [36]; zebrafish: [38]; mammals: [1,2]. These basic comparisons indicate that *akirins *function in a wide range of processes, through direct or indirect regulation of gene transcription, consistent with current literature [1-3,31].

In vertebrates, *akirin1 *is not essential for embryonic development, and has even been lost in the class Aves. Thus, relative to Akirin and Akirin2, Akirin1 has diverged in at least one essential function (i.e. in innate immunity, although other functions of Akirin1 in this system could be masked by functional redundancy [1]). This is supported by significantly faster rates of evolution in multiple sites of Akirin1 compared to its paralogue (fig. 7, fig. 8). However, there were also several sites that have evolved faster in Akirin2 than Akirin1, and could represent regions where a function has been conserved in Akirin1 but was lost in Akirin2. It is known that *akirin1 *(aka *mighty*) has a role in regulating vertebrate myogenesis, as it was identified in mice from a suppression subtraction hybridization cDNA library produced using *myostatin*-null mice as the 'tester' material [3]. Myostatin (aka GDF-8) is a potent negative regulator of mammalian myogenesis and mice lacking a functional copy have a double-muscled phenotype [39]. *akirin1 *was reportedly upregulated in the muscles of *myostatin*^-/- ^mice [3]. Mstn protein was also shown to inhibit the transcription of the *akirin1 *proximal promoter [3]. Interestingly, *akirin1 *also functions in myogenesis in flies. Specifically, Akirin (as Bhringi) bound the bHLH factor Twist and this interaction was necessary for the normal expression of Twist target proteins [32], representing another example of Akirins as co-regulators of transcription. Fly mutants lacking *akirin *had considerable defects in muscle mass and morphology [32]. This is a strikingly opposite phenotype to that induced by the overexpression of *akirin1 *in mdx mice, where muscle mass, fibre size and structural integrity was markedly increased [3]. Thus, the role of mammalian *akirin1 *in regulating muscle growth may be conserved from the *akirin *proto-orthologue. If the function of *akirin1 *in amniote muscle growth is essential, then its absence in birds, where muscle physiology is strongly conserved with mammals, particularly in terms of the functions of key genes (e.g. *myostatin*), could only be fulfilled by *akirin2*.

*akirin2 *(as *FBI1*) was also shown to promote carcinogenesis by interacting with the phosphoserine-threonine-binding protein 14-3-3β [2]. 14-3-3 proteins are highly conserved in eukaryotes and regulate many cellular activities including the cell cycle, intracellular signalling, apoptosis and malignant transformation (reviewed by [35,40]). The 14-3-3β isoform had previously been shown to regulate tumour formation and was upregulated in several cancer cell lines [41] acting through the mitogen-activated protein kinase (MAPK) pathway [42]. *akirin2 *was also upregulated in tumour cell lines and its mRNA downregulation reduced tumour metastasis by inducing the expression of MAP kinase phosphotase 1 (MKP1), which reduced the activation of the extracellular-signal regulated kinases (ERKs), ERK1 and ERK2 [2]. Specifically, the *akirin2*-14-3-3β complex functioned as a transcriptional repressor of the *MKP-1 *promoter [2]. Based solely on the presence of a comparable repertoire of 14-3-3 protein-binding sites, redundancy of this carcinogenic-promoting function with *akirin1 *cannot be excluded. However, distinct evolutionary rates in positions within, or adjacent to 14-3-3 binding sites in Akirin1 and Akirin2 are probably important explanatory variables underlying their functional divergence (fig. 9). Interestingly, there also exists evidence to suggest that *akirin1*, like *akirin2*, indeedfunctions as part of the ERK signalling pathway. It is established that the inhibitory effect of Myostatin on myogenesis is mediated through activation of components of the MAPK/ERK signalling pathway [3,43,44]. *akirin1 *transcription was inhibited by treatment with Myostatin protein and conversely was upregulated by chemical inhibition of MEK1/ERK signalling [3]. Thus, it was suggested that Myostatin signals to *akirin1 *through ERK signalling [3].

In vertebrate immune response signalling pathways, *akirin2 *functions at a level close to, or downstream of NF-κB to selectively regulate some of its target genes [1]. Since a direct interaction of fly Akirin and NF-κB was not demonstrated, it was suggested that Akirins interact with intermediary components [1]. 14-3-3 proteins are potential candidates, since they are known to regulate the nuclear localisation of transcription factors, are found in many transcriptional complexes, can bind to histones and can regulate histone acetylation [35,40]. Importantly, a 14-3-3-Akirin2 complex bound to and regulated promoter activity [2]. 14-3-3 proteins regulate NF-κB activity by binding both IκB and the p65 subunit of NF-kB [45]. IκB is known to inhibit NF-kB by sequestering p65 in the cytoplasm [46] and further, the IκBα isoform also facilitates its nuclear export [47]. TNFα treatment induced the nuclear localisation of 14-3-3 proteins and the disruption of 14-3-3-protein function caused the nuclear localisation of both IκB and p65 [45]. Furthermore, following TNFα treatment, both IκB and 14-3-3β/γ proteins bound to the promoter regions of *IL-6 *and *RANTES*, presumably disrupting the interaction of p65 and chromatin [45]. It was suggested that 14-3-3 proteins formed a complex with IκB and p65 that was efficiently exported from the nucleus [45]. Interestingly, these same NF-KB transcriptional targets (*IL-6*, *RANTES*) were strongly repressed in *akirin2 *knockout mice following TLR, IL-1β and TNFα treatment [1]. Therefore, an interesting line of investigation will be to examine whether the transcriptional repression of NF-kB targets in *akirin2 *knockout mice is accounted for by altered 14-3-3-protein activity. In addition to a predicted interaction with 14-3-3 proteins to regulate chromatin, fly Akirin (as Bhringi) was shown to bind Bap60 [30], a DNA binding protein that forms part of the SWI/SNF-like chromatin remodelling complex [48] which is highly conserved in eukaryotes. Akirin also interacts with the GATA-transcriptional activator Pannier [49] and with TDP45 [30], (TAR DNA binding protein 43), a highly conserved RNA binding protein with roles in transcriptional repression [50] and in regulating exon skipping [51]. It is also noteworthy, that fly Akirin physically interacts with CG1473 [30], a protein with high homology to a E2 Ubiquitin-conjugating enzyme. The ubiquitin-conjugating enzyme UBC13 forms part of the ubiquitin-conjugating complex important in the activation of IKK (and thus activation of NF-κB transcriptional activity) through TRAF6 [52]. CG1473, like Akirin, also binds to the chromatin remodelling protein Bap60 [30], indicating a wider protein-interaction network.

14-3-3 proteins are also known to regulate insulin-like growth factor signalling, a pathway activated by Akirin1 overexpression [3]. The 14-3-3ε isoform binds to phosphorylated forms of both the IGF-I receptor (IGF-I R) and the insulin receptor substrate-I (IRS-I) [53] while the 14-3-3β-isoform binds to activated IRS-I reducing its ability to activate PI(3) kinase (PI(3)K) [54]. During myogenesis, a feed-forward cascade occurs, where IGF-II secreted during early myoblast differentiation, binds to and activates the IGF-IR, in turn activating IRS-1, and the PI(3)k-Akt phosphorylation pathway, which then promotes efficient transcriptional activation of muscle differentiation genes through a MyoD-E-protein complex and several known co-factors [55]. In myoblasts overexpressing Akirin1, differentiation was accelerated, with a concurrent increase in MyoD, Myogenin and IGF-II protein expression, activated Akt expression and a massive increase in the transcription of *IGF*-*II *mRNA [3]. These results suggest that Akirin1 can stimulate IGF-II-PI(3)K-Akt signalling, culminating in the transcription of muscle differentiation genes. Akirin1 has several low affinity 14-3-3 binding sites (fig. 8) and was detected in the cytoplasm [3]. It is therefore possible that the positive effect of Akirin1 on the IGF-II signalling pathway is mediated through binding 14-3-3 proteins in the cytoplasm, sequestering them and effectively stimulating the activation of the IGF1-R and IRS-1 and downstream components of the pathway.

## Conclusion

In summary, the *akirin *gene family is clearly essential to many physiological functions in metazoans and operates in several characterised signalling pathways. This paper provides a necessary evolutionary scaffold to guide future investigations of eukaryote *akirins*. Our exhaustive genomic screens, coupled with the implementation of a common *akirin *nomenclature, should aid researchers in identifying new functions of *akirins *and encourage the propagation of existing research between disciplines. Molecular evolution analyses indicate that vertebrate Akirin1 and Akirin2 proteins have diverged in function and we provide a list of potential underlying candidate residues. An interesting line of future investigation will be to further examine the role played by Akirin-14-3-3 protein interactions in regulating gene expression and signalling cascades in innate immune, myogenic and carcinogenic pathways.

## Methods

### Sequence retrieval

BLASTp searches of the NCBI  non-redundant protein collection using *D. melanogaster *Akirin and *M. musculus *Akirin1/Akirin2 sequences as *in silico *probes, revealed homologues of these proteins in multiple metazoan taxa. Subsequently, manual screening of Ensembl release 50 genome assemblies was performed  using the orthologue and paralogue prediction function with fly *akirin *as a reference point. Ensembl genome assemblies screened included Chordates (from the taxa Ascidiacea, Actinopterygii, Amphibia, Aves, Petromyzontiformes and Mammalia), Arthropods (*Aedes aegypti*, *A*.*gambiae *and *D*.*melanogaster*), nematodes (*C. elegans*) and Fungi (*Saccharomyces cerevisiae*).

To identify *akirin1*/*akirin2 *orthologues in a broader range of metazoans, directed tBLASTn searches of NCBI nucleotide and EST databases were performed for the following taxa: Acoelomorpha, Annelida, Arthropoda, Brachiopoda, Bryozoa, Chaeognatha, Chordata (classes: Ascidiacea, Aves, Cephalaspidomorphi, Cephalochordata and Myxini), Cnidaria, Ctenophora, Echinodermata, Entoprocta, Hemichordata, Mollusca, Nematoda, Nematomorpha, Nemertea, Onychophora, Placozoa, Platyhelminthes, Porifera, Rotifera, Tardigrada and Xenoturbellida. Non-metazoan eukaryotes were also screened by the same approach, including the following taxa: Amoebozoa, Choanoflagellata, Chromalveolata, Fungi, and Plantae. Finally, genome databases at the DOE Joint Genome Institute , Welcome Trust Sanger Institute , Arabidopsis Genome Initiative  and TIGR Rice Genome Annotation  were BLAST screened for *akirin *orthologues for the following taxa: Amoebozoa (*D. discoideum*, *Entamoeba histolytica*), Archea (*Methanococcoides burtonii*,* Sulfolobus islandicus*), Bacteria (*Mycobacterium *sp., *Enterobacter *sp. *Escherichia coli*, *Staphylococcus aureus*), Choanozoa (*Monosiga brevicollis*), Chromalveolata (*Emiliania huxleyi*, *Thalassiosira pseudonana*, *Aureococcus anophagefferens*), Excavata (*N. gruberi*, *Trypanosoma brucei*, *Trichomonas vaginalis, Giardia lamblia*), Fungi (*Aspergillus niger*, *Candida albicans*), Placozoa (*T. adhaerens*) and Plantae (*Chlamydomonas reinhardtii*,* Selaginella moellendorffii*, *Sorghum bicolour*, *Oryza sativa*).

### Comparative analyses of genomic and coding regions

Synteny maps for the genomic neighbourhoods surrounding *akirin1 *and *akirin2 *were constructed using data manually obtained from release 50–52 Ensembl genome assemblies for *H. sapiens*, *M. musculus*, *G. gallus*, *X. tropicalis*, *D. rerio *and *G. aculeatus*. The genomic neighbourhoods surrounding *H. sapiens akirin1*/*akirin2 *were used as a starting reference. The intron-exon organisation of eukaryotic *akirin *orthologues was established by loading genomic and corresponding cDNA sequences into Spidey [56]. PSORTII [34] was used to predict NLSs.

### Phylogenetic analysis of the *Akirin *gene family

27 full coding amino acid sequences of Akirin were used for phylogenetic analysis. This included Akirin1/Akirin2 sequences spanning broad vertebrate taxa as well as deuterostome outgroups representing the single invertebrate gene related to both vertebrate *akirin1*/*akirin2 *in Urochordates (*H. roretzi*), Cephalochordates (*B. floridae*), Hemichordates (*Saccoglossus kowalevskii*) and Echinoderms (*Strongylocentrotus purpuratus*). Sequence alignment was performed using PROMALS [57] at . The first output was improved by removing indels and low scoring regions of the alignment as well as manual checking of alignment quality. ML was performed using PhyML [58] at . The JTT substitution model was utilised with concurrent estimation of the gamma distribution parameter. 1000 bootstrap replicates were sampled to obtain a measure of branch confidence. The Bayesian approach was implemented in MrBayes3.12 [59] with estimation of the substitution rate model, and gamma distribution of among site rate variation. 2 runs were used, each with a single chain of 20 million generations, sampled every 10,000 generations. Convergence was assessed by comparing the standard deviation of split frequencies between runs. 1000 trees were excluded from a total sample of 2001 trees in each run. The independence of the remaining samples was then assessed by analysing autocorrelation in tree log-likelihood values implemented using the ACF function of Minitab 13.2 (Minitab, Inc.). Sample independence was confirmed as no significant increase in log-likelihoods was observed after the burnin phase. Additionally, NJ, ME and MP analyses were performed in Mega 4.0 [60], in each case obtaining branch confidence values by bootstrapping with 1000 iterations. For NJ and ME analyses, the JTT model was used with a gamma distribution parameter estimated by PhyML (α = 0.91). Finally, ASATURA was used to remove saturated amino acid positions from the alignment prior to NJ tree reconstruction [11] using the JTT model.

### Phylogenetic analysis of vertebrate gene families proximal to *akirin1 *and *akirin2*

Phylogenetic analysis was performed on seven vertebrate gene families, where members were represented on both *akirin1 *and *akirin2 *containing chromosomal tracts in at least two vertebrate classes (further details are provided in the results and fig. 7). High quality amino-acid translations were obtained from Ensembl release 52 genome databases for representatives of four vertebrate taxa (mammalia, aves, amphibia and Actinopterygii). Outgroup sequences were obtained either through orthologue screening of Ensembl databases for *C. intestinalis*, or non chordate invertebrates, or by BLAST screening of NCBI *C. intestinalis *or *B. floridae *protein databases. Sequence alignment was performed with Promals [57] followed by manual checking and submission to Gblocks at  to remove poorly aligned and divergent regions [61]. Bayesian phylogenetic reconstruction was performed as for the Akirins, except with different sampling parameters for each gene family. Briefly, 5 million generations were performed with sampling every 2500 generations for the ras-related GTP-binding, heterogeneous nuclear ribonucleoprotein, cytosolic 5'-nucleotidase 1, proline-rich nuclear receptor coactivator, glycoprotein endo-alpha-1,2-mannosidase families. For the cannabinoid receptor and potassium voltage-gated channel families, 10 million generations were performed with sampling every 5000 generations. In each analysis, runs had converged (i.e. the standard deviation of split frequencies between runs was <0.005) before half of the final number of generations were reached. 1000 trees were excluded from a total sample of 2001 trees in each run before consensus phylogenies were reconstructed. ML, NJ and MP analysis were performed essentially as described for the Akirin dataset.

### Tests of selection and rate shifts

Estimates of synonymous and non-synonymous substitution rates for Akirin1 and Akirin2 were performed using codon-alignments obtained by loading aligned amino acid and corresponding nucleotide sequences into PAL2NAL [62]. Akirin1 orthologues from *H. sapiens*, *M. musculus*, *X. tropicalis *and *D. rerio *were compared. Akirin2 orthologues from *H. sapiens*, *M. musculus*, *G. gallus*, *X. tropicalis *and *D. rerio *were compared. PAL2NAL was set to automatically calculate synonymous and non-synonymous substitution rates, for each pairwise comparison using a model [26] normally implemented in codeml of PAML [63]. Additionally, two codon alignments were produced separately for the Akirin1 and 2 orthologues described above and loaded into Mega 4.0 [60]. Pairwise estimates of the number of synonymous and non-synonymous substitutions between different orthologues were then calculated using the Nei-Gojobori method [27], with the P-distance model.

To examine potential shifts in evolutionary rates between Akirin paralogues, an amino acid alignment with 14 Akirin2 orthologues, 9 Akirin1 orthologues and 4 Akirin orthologues from invertebrate deuterostomes (additional file 2) was loaded into DIVERGE [33] with a corresponding phylogenetic tree in Newick format, that had the topology obtained by ML (fig. 3). The Akirin1 and Akirin2 clades were defined as separate clusters and the coefficient of functional divergence and posterior probability for functional divergence at each site in the alignment were estimated using the Gu99 algorithm [28]. Additionally, the same alignment was loaded into the rate shift analysis server at [29] along with the same Newick file. Akirin1, Akirin2 and Akirin (outgroup) clusters were defined and the JTT model was employed.

## Authors' contributions

DJM performed all experiments and produced all figures. Both authors conceived the study and wrote, edited and approved the manuscript.

## Supplementary Material

Additional file 1 **Detailed information on *akirin *orthologues identified in a broad range of metazoans.** The table provides exhaustive data on *akirin *sequences identified in a screen of genome and transcriptome databases for 22 metazoan phyla. It should be noted that the absence of a sequence from specific lineages does not, in most cases, distinguish between artefacts of insufficient sequencing data and gene non-functionalization. Click here for file

Additional file 2 **Alignment of Akirin proteins used for evolutionary analyses.** The file, which is in Fasta format, is the amino acid alignment of Akirin sequences used for phylogenetic reconstructions, the DIVERGE analysis and the likelihood ratio test. Accession numbers and other identifiers are shown next to each sequence. Click here for file

Additional file 3 **Protein alignments of seven gene families found in chromosomal proximity to *akirin1 *and *akirin2*.** The text file provides Fasta format amino acid alignments used for phylogenetic reconstructions of seven gene families that were located in the genomic neighbourhood of both *akirin1 *and *akirin2 *in multiple vertebrate classes. Sequences were mainly obtained from Ensembl release 52 genome databases and are shown with gene identifiers and their chromosomal location. Alternatively, NCBI accession numbers are provided. Click here for file

Additional file 4 **Pairwise synonymous and non-synonymous substitution rates for Akirin1 and Akirin2 orthologues.** The table provides separate estimates of pairwise synonymous (dS) and non-synonymous (dN) substitution rates for Akirin1 and Akirin2 orthologues from mammals, birds, amphibians and teleost fishes as performed by two methods (see main text). Also shown are the accompanying dN/dS ratios. Click here for file

Additional file 5 **Amino acid alignment of 27 Akirin sequences showing the full results of the rate-shift likelihood ratio test.** This file shows an output from the rate-shift analysis server  where the LRT (ref [29] in the main text) was performed. Sites highlighted as red or blue in both Akirin1 and Akirin2 have evolved at a respective faster and slower rate for both paralogues, relative to the average for all positions in the alignment. Sites highlighted in either red or blue in one Akirin paralogue or the other were calculated to have evolved at a respective faster or slower rate in one paralogue and are likely to have contributed to functional divergence after the Akirin duplication. Alignment name abbreviations are: P.m (*P. marinus*), H.s (*H. sapiens*), X.t (*X. tropicalis*), D.r (*D. rerio*), T.r (*Takifugu rubripes*), G.g (*G. gallus*), G.a (*G. aculeatus*), O.l (*Oryzias latipes*), P.p (*P. promelas*), S.a (*Sparus aurata*). Click here for file
