# Supplementary material for: Evolution of the multifaceted eukaryotic akirin gene family
Source: BMC Evol Biol. 2009 Feb 6;9:34. doi: 10.1186/1471-2148-9-34 (PMC2660306; doi:10.1186/1471-2148-9-34)
Supplement: Additional file 5 — Amino acid alignment of 27 Akirin sequences showing the full results of the rate-shift likelihood ratio test. This file shows an output from the rate-shift analysis server where the LRT (ref [29] in the main text) was performed. Sites highlighted as red or blue in both Akirin1 and Akirin2 have evolved at a respective faster and slower rate for both paralogues, relative to the average for all positions in the alignment. Sites highlighted in either red or blue in one Akirin paralogue or the other were calculated to have evolved at a respective faster or slower rate in one paralogue and are likely to have contributed to functional divergence after the Akirin duplication. Alignment name abbreviations are: P.m (P. marinus), H.s (H. sapiens), X.t (X. tropicalis), D.r (D. rerio), T.r (Takifugu rubripes), G.g (G. gallus), G.a (G. aculeatus), O.l (Oryzias latipes), P.p (P. promelas), S.a (Sparus aurata). [file 1471-2148-9-34-S5.pdf]

```
                    1                                                                              80
D.r(Akirin1)        MACGATLKRS MEFE-ALLPQ SPKRRRCNPL PGTPTTPSPQ RCSLRPSTES PAH---SMSP QSMGGEHRLT -PEQIFQNIR
P.m(Akirin1)        MACGATLKRQ LDFEPLLSSQ SPKRRRCTPL SPPSPTSSGY LQAEPTASLG ESHNYH-LHS PAAAA---RL TPEQVLANLK
P.p(Akirin1)        MACGATLKRS MEFE-ALLPQ SPKRRRCNPL PGTPTTPSPQ RCGLRPSTES PAH---SMSP QSMAGEHRLT -PEQIFQNIR
T.r(Akirin1)        MACGATLKRS MEFE-ALLPQ SPKRRRCNPL PGTPSTPSPQ RCNLRPPVDS PTH---SMSP SAIGGEHRLT -PEQIFQNLR
G.a(Akirin1)        MACGATLKRS MEFE-ALLPQ SPKRRRCNPL PGTSGTPSPQ RCNLRPPVDS PAH---SMSP PAMGGEHRLT -PEQIFQNLR
O.l(Akirin1)        MACGATLKRS MEFE-ALLPQ SPKRRRCNPL PGTPSTPSPQ RCNLRPPIDS PTH---SMSP QPIGGEHRLT PVEQIFQNLR
X.t(Akirin1)        MACGATLKRS MEFE-ALMPQ SPKRRRCAPL PGSPATPSP- QRCAIRPEMQ QGQ---QQPL SQLGGDRRLT -PEQILQNIK
M.m(Akirin1)        MACGATLKRP MEFEAALLPG SPKRRRCAPL PGPTPGLRPA EPPPLQMQTP PAS---LQQP APPGSERRLP TPEQIFQNIK
H.s(Akirin1)        MACGATLKRP MEFEAALLPG SPKRRRCAPL PGPTPGLRPE PPPPFQTQTP PQS---LQQP APPGSERRLP TPEQIFQNIK
H.s(Akirin2)        MACGATLKRT LDFDPLLSPA SPKRRRCAPL SAPTSAAASA TAASFSAAAA SPQKYLRMEP SPFGDVSSRL TTEQILYNIK
M.s(Akirin2)        MACGATLKRT LDFDPLLSPA SPKRRRCAPL SAPASAAASA AAA--SAAAA SPQKYLRMEP SPFGDVSSRL TTEQILYNIK
G.g(Akirin2)        MACGATLKRT LDFDPLLSPA SPKRRRCAPL SAPASAAAS- -----SSSFA SPQKYLRMEP SPFGEVSSRL TTEQILYNIK
X.t(Akirin2)        MACGATLKRT IEFDPLLSAA SPKRRRCAPL SPP------- --------GP SPQKYLRMEP SPFGEVSPRL TAEQILYNIK
P.p(Akirin2[1])     MACGATLKRT MDFDPLLNQA SPKRRRCAPL SPSSS----- --------SA SPQKYLRMEP SPFGRVSSAL TTEQILSNIK
D.r(Akirin2[1])     MACGATLKRT MDFDPLMNPA SPKRRRCAPM SPSSS----- --------SA SPQKYLRMEP SPFGRVSSAL TTEQILSNIK
G.a(Akirin2[1])     MACGATLKRT LDFDPLMSQA SPKRRRCAPI ASP------- --------VS SPQKYSRMEP SPFGEVSSRL TTEQILHNIK
S.a(Akirin2[1])     MACGATLKRS LDFDPLMNPA SPKRRRCAPI MSP------- --------VS SPQKYLRMEP SPFGEVSSRL TAEQILHNIK
T.r(Akirin2[1])     MACGATLKRT LDFDPLMNPA SPKRRRCTPM MSP------- --------VS SPQKYLRMEP SPFGEVSSRL TTEQILHNIK
O.l(Akirin2[1])     MACGVTLKRT LDFDPLMNQA SPKRRRCTPV MSP------- --------VS SPQKYLRMDP SPFGEVSSRL TTEQILHNIK
S.a(Akirin2[2])     MACGATLKRT MDFDPLMSPT SPKRRRCIPV SPSSS----- --------SS SPRKYLSMEP SPFGESSSTL SAEQILNSIK
T.r(Akirin2[2])     MACGATLKRT MDFDPLMSPT SPKRRRCVPV STS------- --------SP SPRKYLRMEP SPFGEFSSTI SAEQILQSIK
O.l(Akirin2[2])     MACGATLKRS MDFDPLTNPT SPKRRRCIPV TPS------- --------SA APRKYLRMEP SPFGESSSKL SLEQILHSIK
G.a(Akirin2[2])     MACGATLKRT MDFDPLMSPT SPKRRRCIPV SHSSS----- --------SS SPRKYLSMEP SPFGESSSRL SAEQILNSIK
Echinoderm          MAC-ATLKRT LDFDPLHSGT SPKRRRCSPI ITPSTPTS-- ---------- ----KSSLQQ SPFSPVTPRI SPGQLVAHIS
Cephal...ordate     MAC-ATLKRT YELDPLHSRP SKRPRRCMPM AGHSPNVSPT ---------- ----PPRQKP SSFPEVEPKL TSEQISQFIR
Urochordate         MACGATLKRS LDFDPLHAVS PNKRRRLSPR SGSVYTLGEI STL------- ----QRSPPS SPFKNSAQTL SSGQILKNIY
Hemichordate        MACGATLKRT LDFDPLHSSP SPKRRRCVPI IPP------- --------TT PQTKAHKLEP SPFGQVSPKL TPEQLSNSVL

                    81                                                                             160
D.r(Akirin1)        QEYSRYQRRR QLEGAFNQTE PCSSTDIQSS SPALTSPTGA SSLKKDQPLF TLRQVGYLCE RLIKDHEEKM REEYEQILNT
P.m(Akirin1)        QEYSRMQRRL QVDGPPDA-- -------HAGD SASMQSGVSG DGGRREQPLF TLRQVGLICD GLLREREVHI RQEYDQILST
P.p(Akirin1)        QEYSRYQRRR QLEGAFNQTE PCSSTDIQSS SPALTSPTGA STLKKDQPLF TLRQVGYLCE RLIKDHEEKM REEYEQILNT
T.r(Akirin1)        QEYSRIQRRR QLEGAFNQTE ACSSSDAPSP SSSLHAPPPA GASRKDQPSF TLKQVSYLCE RLLKDHEEKI REEYEQILNT
G.a(Akirin1)        QEYSRIQRRR QLEGAFNQTE ACSSSDSPSP SSSINAPSPP GASRKDQPSF TLKQVSYLCE RLLKDHEEKI REEYEQILNT
O.l(Akirin1)        QEYSRIQRRR QLEGAFNQAE ACSSSDAPSP SSSLTAPSPP GASRKDQPSF TLRQVSLLCE RLLKDHEEKI REEYEQILNT
X.t(Akirin1)        QEYTRYQRRR QLEGAFNQCE AGALNEVQAS CSSLTAPSPG SLVKKDQPTF SLRQVGILCE RLLKDHEDKI REEYEQILNI
M.m(Akirin1)        QEYNRYQRWR HLEVVLSQ-S EACTSETQPS SSALTAPPGA FWMKKDQPTF TLRQVGIICE RLLKDYEDKV REEYEQILST
H.s(Akirin1)        QEYSRYQRWR HLEVVLNQ-S EACASESQPH SSALTAPPGS SWMKKDQPTF TLRQVGIICE RLLKDYEDKI REEYEQILNT
H.s(Akirin2)        QEYKRMQKRR HLETSFQQTD PCCTSDAQPH AFLLSGPAAS SPLKKEQPLF TLRQVGMICE RLLKEREEKV REEYEEILNT
M.s(Akirin2)        QEYKRMQKRR HLEASFQQAD PGCTSDSQPH AFLISGPATS SPLKKEQPLF TLRQVGMICE RLLKEREEKV REEYEEILNT
G.g(Akirin2)        QEYKRMQKRR HLENSFQQTD PCCSTDAQPH AFLLTGPAAS SPLKKEQPLF TLRQVGMICE RLLKEREEKI REEYEEILTT
X.t(Akirin2)        QEYKRMQKRR HLEASFQPTD PCCSSEGQPQ TFIPSGPSAT SPLRKEQPLF SLRQVGMICE RLLKEREDKV REEYEEILTT
P.p(Akirin2[1])     QEYKRMQKRR HLESSFQQ-- ---SESVCGL ESGPHASGAV SPSRREQPLF TLKQVGMICE RLLKEREEKV REEYDEILTT
D.r(Akirin2[1])     QEYKRMQKRR HLESSFQQ-- --TDSCCPLD SQPHASSGSA SPSRREQPLF TLRQVGMICE RLLKEREEKV REEYDEILTT
G.a(Akirin2[1])     QEYKRLQKRR HLDSGFQQ-- -ADSCCPLDL QNVHSGPGAS SPTRKEQPLF SLRQVGMICE RLLKEREDKI REDYDEMLTT
S.a(Akirin2[1])     QEYKRLQKRR HLDSAFQQ-- -ADSSCPVDL QNIYSGSGAS SPTRKEQPLF SLRQVGMICE RLLKEREDKI REEYDEILTT
T.r(Akirin2[1])     QEYKRLQKRR HLDNAFQQAD GCCPLDLQSI PSCLALPAAT SPSRKEQPLF SLRQVGMICE RLLKEREDKI REEYDEILTT
O.l(Akirin2[1])     QEYKRLQKRR HLDSTFLQ-- -SDGCCPVDL QSSQCGSAAS PPSRKEQPLF SLRQVGMICE RLLKEREDKI REEYDETLTT
S.a(Akirin2[2])     QEYKRIQKRK HLDGGYQQSE CCYSPESPSQ SSTMNVSGGV SPTRKEQPLF TLRQVGMICE RLLKEREEKV REEYEETMTS
T.r(Akirin2[2])     QEYKRIQKRK HLDGGYLQSD CSYSPESPSQ SSSMHISGGV SPTRKEQPLF TLRQVGIICE RLLKEREEKV REEYEETMTS
O.l(Akirin2[2])     QEYKRIHKRK HLDGSYHQ-- ---SECCYSP ESPSQSSGGV SPTRKEQPLF TLRQVSMICE RLLKEREEKV REEYEETMTS
G.a(Akirin2[2])     QEYKRIQKRK HLDSGYQPSE GCYSPESPSQ SSTMTVSGGV SPTRKEQPLF TLRQVGMICE RLLSEKEETV REEYEETMTS
Echinoderm          HEWRRIRRRK RLEESYSSEA PSSSEMVETS GCFSPPPSLQ QTQTKDQPMF TLKQVILICE QMLKEQESRI REEYDKALSC
Cephal...ordate     DEYRRMHRRR RLWGATDSNV QSAASPSHHG SLSPMSPGTQ SPINKDKPLF SMRQVSMICE RMIKERDTQV REEYDKVLSC
Urochordate         DEYKKMKHRK QLMMNPQAPH DLASNVPSAS SFLLSQPPQT EKKITETPLL TMKQVGLICE RLLKEQEEKL RSEYEKALVA
Hemichordate        LEYKRMQRRK KLATAFTSGT STYPAPGPSH QQQQSAAPSS SPSRREQPIF TLRQVIMLCE RLLKEREEQI KAEYDKTLTC

                    161                          190
D.r(Akirin1)        KLAEQYESFV KFTQDQIMRR YGARPASYVS
P.m(Akirin1)        KLAEQYEGFV KFTQDQIMRK YGERPASYVS
P.p(Akirin1)        KLAEQYESFV KFTQDQIMRR YGARPASYVS
T.r(Akirin1)        KLAEQYESFV KFTQDQIMRR YGARPASYVS
G.a(Akirin1)        KLAEQYESFV KFTQDQIMRR YGARPASYVS
O.l(Akirin1)        KLAEQYESFV KFTQDQIMRR YGARPASYVS
X.t(Akirin1)        KLAEQYESFV KFTHDQIMRR YGARPASYVS
M.m(Akirin1)        KLAEQYESFV KFTHDQIMRR YGTRPTSYVS
H.s(Akirin1)        KLAEQYESFV KFTHDQIMRR YGTRPTSYVS
H.s(Akirin2)        KLAEQYDAFV KFTHDQIMRR YGEQPASYVS
M.s(Akirin2)        KLAEQYDAFV KFTHDQIMRR YGEQPASYVS
G.g(Akirin2)        KLAEQYDAFV KFTHDQIMRR YGEQPASYVS
X.t(Akirin2)        KLAEQYDAFV KFTHDQIMRR FGEQPASYVS
P.p(Akirin2[1])     KLAEQYDAFV KFTHDQLMRR FGEQPASYVS
D.r(Akirin2[1])     KLAEQYDAFV KFTHDQLMRR FGEQPASYVS
G.a(Akirin2[1])     KLAEQYDAFV KFTHDQLMRR FGEQPASYVS
S.a(Akirin2[1])     KLAEQYDAFV KFTHDHWMRR FGEQPASYVS
T.r(Akirin2[1])     KLAEQYDAFV KFTHDQLLRR FEEQPASYVS
O.l(Akirin2[1])     KLAEQYDAFV KFTHDQLMRR FGEQPASYVS
S.a(Akirin2[2])     KLAEQYDTFV EFTHDQLMRR FGEQPASYVS
T.r(Akirin2[2])     KLAEQYDTFV KFTHDQLMRR FGEQPASYVS
O.l(Akirin2[2])     KLAEQYDTFV KFTHDQLMRR FGEQPASYVS
G.a(Akirin2[2])     KLAEQYDTFV KFTHDQLMRR FGEQPASYVS
Echinoderm          KLAEQYDAFV KFNQDQIQRR FTSTSMSYVS
Cephal...ordate     KLAEQYDAFI RFNQDYLQRR FAETAASYVS
Urochordate         KLSEQYDAFV KFNYDQVQRR ISEHPASYVS
Hemichordate        KLAEQYEAFL KFNHDQLHRR FGETAASYVS
```
